# Sperm BerserKers

**DOI:** 10.7554/eLife.01469

**Published:** 2013-10-08

**Authors:** David E Clapham

**Affiliations:** 1 **David E Clapham** is at the Howard Hughes Medical Institute, Department of Cardiology, Boston Children’s Hospital, Boston, United States, and the Department of Neurobiology, Harvard Medical School, Boston, United Statesdclapham@enders.tch.harvard.edu

**Keywords:** Human KSper, Slo1, spermatozoa, sperm ion channels, Big Potassium (BK) channel, CatSper, Human, Mouse

## Abstract

Human sperm cells rely on an unusual type of potassium ion channel.

**Related research article** Mannowetz N, Naidoo NM, Choo S-AS, Smith JF, Lishko PV. 2013. Slo1 is the principal potassium channel of human spermatozoa. *eLife* **2**:e01009. doi: 10.7554/eLife.01009

**Image** Increasing the concentration of progesterone reduces the current through the potassium ion channel in human sperm (red line) and eventually blocks the channel (blue)

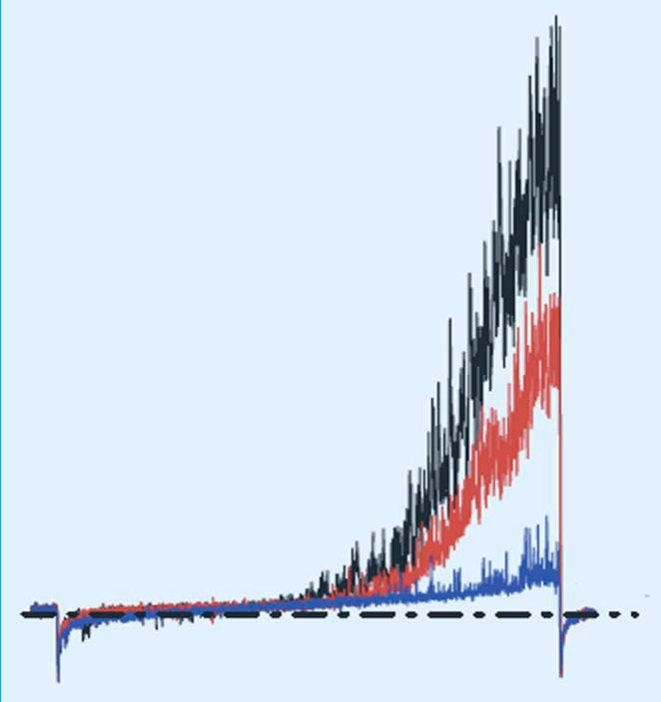

Sperm have an unusual job description: leave one organism, invade another, and deliver their DNA into a host cell. Like infectious agents, many try but few succeed, with each sperm having the same chances of success as you or I have of winning a national lottery. Nonetheless, against steep odds, there are more than seven billion human lottery winners alive today.

In recent years, our understanding of how mammalian sperm do their job has been greatly improved as a result of patch clamp experiments in which glass electrodes provide researchers with electrical control over the membranes of individual sperm cells and access to the interiors of these cells (Kirichok et al., 2006). These experiments have revealed the central role played by the ion channels that control the flow of sodium, calcium, potassium and protons into and out of many types of cells. Now, in *eLife*, Polina Lishko of the University of California at Berkeley and colleagues—including Nadja Mannowetz as first author—show that the mechanism used to control of the flow of potassium out of human sperm cells is different to that in mouse sperm cells (Mannowetz et al., 2013).

If we disregard their relatively small heads, sperm are basically ATP-driven motors whose movement resembles that of sea snakes. Without guidance, however, they just spin in circles (Miki and Clapham, 2013). In mammals, this guidance is provided by the walls of the female reproductive tract, by vaginal secretions that flow gently along the oviduct, and by the increasing alkalinity of these secretions as sperm ascend from the vagina to the egg. In humans, there is an additional cue in the form of the female hormone progesterone, which is thought to come from the cells surrounding the egg (Lishko et al., 2011; Strunker et al., 2011). All these cues lead to large numbers of calcium ions entering the sperm cell and, ultimately, to the tail undergoing a vigorous whip-like thrashing motion. This is called hyperactivated motility and successful fertilization cannot happen without it (Yanagimachi, 1994).

So what is the mechanism that excites sperm into this berserker mode? The answer is the entry of calcium ions through an ion channel called CatSper that is only found in sperm (Ren et al., 2001; Carlson et al., 2003). The CatSper channel is present in many species, from water mould to Silvio Berlusconi, and it is the most complex ion channel known (Chung et al., 2011).

Despite its complexity, CatSper’s job is pretty simple: all it has to do is to open and allow calcium ions to flow into the sperm tail—the very steep calcium concentration gradient (10,000 times higher inside the cell than outside) does most of the work. Once the calcium ions enter the tail, they initiate an unknown and seemingly complex cascade of phosphorylation events that, as described above, result in the hyperactivated motility that is an essential prerequisite of successful fertilization (Visconti et al., 1995). If any of the CatSper genes is faulty, mice or men are infertile (Qi et al., 2007; Smith et al., 2013).

Mannowetz, Lishko and colleagues—including Natasha Naidoo and Seung-A Sara Choo from Berkeley, and James Smith from UCSF—focus their attention on the potassium channel in human sperm and show that it is a member of the Slo family of ion channels. The potassium channels in mouse sperm have been extensively studied and they are known to be Slo3 channels (Zeng et al., 2011). However, Mannowetz et al. find that, unlike the Slo3 potassium channels in mice, the potassium channels in human sperm are not sensitive to pH. Second, the potassium channels in human sperm are blocked by three toxins (charybdotoxin, iberiotoxin, and paxilline) that have no impact on mouse sperm. Third, Western blotting detects Slo1 in human sperm. Mannowetz et al. conclude, therefore, that the principal potassium channel in human sperm is Slo1, also known as known as *KCNMA1* or BK, among other things.

All this nomenclature aside, all one has to remember is that potassium ions leave a human sperm cell via the Slo1 channels when the concentration of calcium ions inside the cell increases. This makes the electrical potential of the cell membrane more negative (a process called hyperpolarization), which leads to even more calcium ions entering the cell. Like the volume knob on a radio, the potassium channels control the level of calcium ions inside the cell by controlling the electrical forces that drive them into the cell. The potassium channels are able to influence the calcium ions because each of the four subunit of the Slo1 channel in humans has two domains that can bind calcium ions (Wu et al., 2010).

There is one nagging worry. Many phenotypes—including abnormal hearing, vascular function, gait and urination—have been associated with Slo1 mutations, but problems with human male fertility have not, so far, been associated with Slo1 mutations. However, since there are many causes for male infertility, and since in vitro fertilization ‘cures’ this problem, genes for male infertility are rarely hunted and identified. In contrast, it is known that deletion of the gene for Slo3 causes infertility in male mice (Zeng et al., 2011). All this raises two questions: Did the enormous evolutionary pressure on eggs and sperm switch the potassium channel in human sperm from Slo3 to Slo1 to accommodate activation by progesterone rather than alkalinization? And do the various accessory subunits found in Slo1 fine-tune the behaviour of potassium channels in humans? We have not heard the last of this interesting story. My prediction? There will be eight billion lottery winners by 2020.

**Figure 1.**
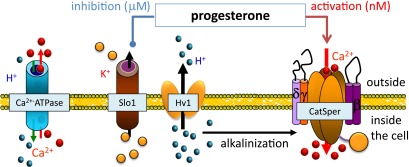
Ion channels work together to ensure that sperm cells are hyperactivated. Sperm cells contain a variety of ion channels that control the movement of ions and protons (H^+^) into and out of the cell (Mannowetz et al., 2013). As a sperm cell moves up the fallopian tube, the CatSper ion channel (right), which controls the movement of calcium ions (Ca^2+^), is partially activated as a result of alkalinization inside the cell (caused by protons leaving through the Hv1 ion channel) and low levels of progesterone outside the cell. As the sperm gets closer to the egg, the increased levels of progesterone inhibit the Slo1 ion channel, causing potassium ions (K^+^) to leave the cell. This hyperpolarizes the cell membrane and leads to full activation of the CatSper ion channel. The resulting influx of large numbers of calcium ions leads to hyperactivation of the sperm—the vigorous tail thrashing motion that is a prerequisite of fertilization. Protons and calcium ion can also move through the Ca2+ ATPase transporter (left).
